# One Year of COVID-19 Pandemic in Italy: Effect of Sedentary Behavior on Physical Activity Levels and Musculoskeletal Pain among University Students

**DOI:** 10.3390/ijerph18168680

**Published:** 2021-08-17

**Authors:** Federico Roggio, Bruno Trovato, Silvia Ravalli, Michelino Di Rosa, Grazia Maugeri, Antonino Bianco, Antonio Palma, Giuseppe Musumeci

**Affiliations:** 1 Department of Psychology, Educational Science and Human Movement, University of Palermo, Via Giovanni Pascoli 6, 90144 Palermo, Italy; federico.roggio@unipa.it (F.R.); antonino.bianco@unipa.it (A.B.); antonio.palma@unipa.it (A.P.); 2 Department of Biomedical and Biotechnological Sciences, Human, Histology and Movement Science Section, University of Catania, Via S. Sofia n°87, 95123 Catania, Italy; brunotrovato94@gmail.com (B.T.); silviaravalli@gmail.com (S.R.); mdirosa@unict.it (M.D.R.); graziamaugeri@unict.it (G.M.); 3 Research Center on Motor Activities (CRAM), University of Catania, 95123 Catania, Italy; 4 Department of Biology, College of Science and Technology, Temple University, Philadelphia, PA 19122, USA

**Keywords:** COVID-19, physical activity, sedentary behavior, university, musculoskeletal pain, neck pain, low back pain, health prevention

## Abstract

The COVID-19 outbreak forced Italian students to reduce their daily activities, inducing a seden-tary attitude that was worsened by distanced learning. This study aimed to survey the physical activity levels that were maintained before and during the social restrictions following the pan-demic, their correlation to musculoskeletal pain, as well as analyzing the impact of these COVID-19 restrictions on pain and fatigue that affects daily life activities. A total of 2044 students completed the online questionnaire, of which the results of 1654 participants were eligible. Before the pandemic, the levels of physical activity were distributed as: 19.9% no activity, 30.1% light ac-tivity, 21.5% moderate activity, and 28.5% high activity. After one year of the pandemic, 30.6% of the participants were inactive, 48.1%, 10.9%, and 10.5% stated as maintaining, respectively, light, moderate and high levels of physical activity. Furthermore, 43.5% reported neck pain and 33.5% stated to experience low back pain. Physical activity levels lower than 150 min/week may have predisposed students to suffer from neck pain (1.95 OR at 95% CI, 1.44–2.64) and low back pain (1.79 OR at 95% CI, 1.29–2.49). A positive correlation between physical activity levels, Verbal Descriptive Scale (VDS), and pain frequency have been observed for neck and low back pain (*p*-value < 0.05). Finally, low physical activity levels were associated with musculoskeletal pain onset and pain worsening.

## 1. Introduction

The severe acute respiratory syndrome coronavirus 2 (SARS-CoV-2), responsible for the coronavirus disease 2019 (COVID-19), has spread worldwide since the first reported case in Wuhan in late December 2019, becoming the primary threat to public health in all countries. Since the World Health Organization (WHO), on 11 March 2020, declared the COVID-19 pandemic, many countries, including Italy, launched public health security plans based on the promotion of social distancing, wearing anti-infection masks, and lockdown restrictions. These extreme measures induced lifestyle changes; in particular, quarantine caused a reduction in physical activity (PA) levels per week in all different age groups, leading to decreased levels of psychological well-being in Italy [1]. Physical inactivity also plays a crucial role in non-communicable chronic disease, which is considered responsible for over three million premature deaths worldwide every year [2]. The relationship between quarantine-imposed reduced PA levels and musculoskeletal pain (MP) is a topic of growing interest today. Sagát et al. [3] showed how this extreme lifestyle change led to an increase in low back and neck pain prevalence in Riyadh’s population aged 18 to 64. Toprak et al. [4] compared people who stayed home and continued to work during the three-month lockdown of the pandemic in Turkey, and found that those who stayed home had increased MP symptoms that were likely due to lower PA levels.

One of the social categories most affected by restricted mobility and reduced PA levels is students. Students typically spend many hours seated on non-ergonomic chairs and assuming incorrect postures to carry out their curricular activities, leading to a general musculoskeletal overload [5]. Furthermore, while spending much time using laptops and smartphones to study and support leisure activities, they adopt incorrect postures, leading to musculoskeletal alteration and pain, especially to the neck and the spine [6]. Haroon et al. [7] reported a high incidence of MP in Karachi’s university medical students, identifying the usage of laptops for more than three hours per day as a risk factor for neck pain. Different studies [8,9,10] conducted among university students highlighted a high percentage of them stating MP, especially at the neck and low back. Furthermore, inactive students commonly reported mild/moderate pain, while physically active students referred to severe pain with a higher frequency over the month than inactive ones.

This study aimed to survey the PA levels and their correlation to MP among university students from Italy, before and during the pandemic restriction, therefore analyzing the impact of COVID-19 restrictions on pain and fatigue affecting daily life activities.

## 2. Materials and Methods

### 2.1. Design 

This study employed an online survey addressed to Italian university students through the Google Forms web survey platform (Google LLC, Mountain View, CA, USA). The online survey was displayed through social media, such as Instagram and Facebook, and sent to personal contacts and university students via WhatsApp and email. Participants were informed about the aims of the study, data anonymization, and protection. They were asked to provide informed consent before participation: “The data of this survey are anonymous, and their confidentiality will be guaranteed in compliance with the Italian and European legislation on the protection of personal data. We remind you that participation is voluntary, and therefore you can withdraw or give up at any time. The participant, adequately informed of the methods and purposes of the research described above, gives his consent to his participation, ensuring that his personal data will be treated anonymously”. 

### 2.2. Participants 

A total of 2044 Italian university students completed the online Google form questionnaire from 8 February to 21 March 2021, during the COVID-19 lockdown in Italy. A data cleansing process has been applied to remove ineligible data following the American Association for Public Opinion Research guidelines [11]. The eligible responses for this study were 1654: 1026 from females (62%) and 628 from males (38%).

### 2.3. Questionnaire

The online questionnaire (https://forms.gle/dzCy8MSdYUdq3wEb8) (accessed on 8 February 2021) aimed to investigate the effect of a sedentary lifestyle and the presence of spinal pain due to the pandemic restrictions among university students. The questionnaire was divided into four sections. The first (1) section comprised demographic, anthropometric, socioeconomic data, health status, and lifestyle questions. In addition, it introduces questions concerning PA levels before and during the COVID-19 pandemic, whether weight gain occurred during one year of restrictions, and how many hours were spent seated during the day due to online distance learning. The second (2) and third (3) sections comprised cervical and lumbar questions investigating whether the students ever experienced spine pain before the pandemic, or if the pain occurred for the first time during the pandemic, pain score based on Verbal Descriptive Scale (VDS) [12], pain frequency during the week/month. Furthermore, the moment of the day and the type of posture in which the pain occurs, symptoms related to the pain, and if they use drugs or specific exercise to reduce the pain. The fourth (4) section was an adapted version of the SF-36 “limitation of activities” section [13] which investigated the perception of breathlessness, i.e., air hunger, tachycardia, perceived during the COVID-19 emergency (from March 2020 to March 2021) due to anti-infection mask usage. This section comprised five questions with three possible answers, each related to a specific score: no reduction = 2, mild reduction = 1, and severe reduction = 0. Based on the SF-36 recommendations for scoring protocol, participants of the study achieving a high overall score were classified as no fatigue subjects; meanwhile, those with a low overall score were classified as moderate fatigue subjects, according to the SF-36 classification method. 

### 2.4. Statistical Analysis

Statistical analysis was performed using R Project for Statistical Computing (Vienna, Austria) [14,15] and Jamovi (Version 1.6, Sydney, Australia) [16]. The analysis was conducted with the use of descriptive statistics. Statistical differences between groups were tested using the *t*-test, Chi-square test, and the Kendall–tau rank correlation coefficient. The odds ratio (OR) and corresponding 95% confidence intervals (CI) were calculated. Levels of significance were set at α < 0.05. 

## 3. Results

### 3.1. General Characteristics of the Study Population

Baseline characteristics of the study subjects are displayed in Table 1. Overall, the study sample (*n* = 1654) comprised 62% females and 38% males. The sample consists of university students whose mean age was 22.51 ± 3.12 (*p*-value < 0.05), height 169.04 ± 8.84 centimeters, weight 65.24 ± 13.38 kg, and body mass index (BMI) was 22.7 ± 3.5 kg/m^2^. The values of BMI identified three categories: underweight (BMI < 18.5), normal weight (BMI 18.5–24.9), and overweight (BMI > 25.0), 71.1% of participants were classified as normal-weight subjects.

### 3.2. Physical Activity Levels before and after One Year of the COVID-19 Pandemic in Italy

Participants gave information about PA levels before the pandemic, i.e., before March 2020, and during the pandemic, i.e., from March 2020 to March 2021, considering whether the pandemic had increased or decreased PA. The subjects were therefore clustered into four categories depending on their level of PA per week: no activity (0 min/week), light activity (<140 min/week of PA), moderate activity (≃150 min/week of PA), and high activity (>200 min/week of PA). Before the pandemic, the total number of participants is distributed according to activity levels: 19.9% no activity, 30.1% light activity, 21.5% moderate activity, and 28.5% high activity. During one year of pandemic restriction, the total number of participants is distributed according to activity levels: 30.6% no activity, 48.1% light activity, 10.9% moderate activity, and 10.5% high activity, Table 2. The data report an increase of no activity behavior and a drastic decrease of moderate and high PA levels following one year of pandemic. However, the percentage of those practicing light levels of PA recorded an increase of 18%. 

**Figure 1:**
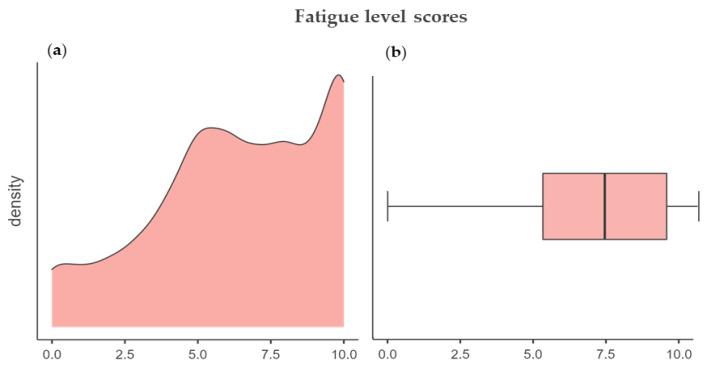
(**a**) A density plot showing the concentration of scores and (**b**) a boxplot showing the difference of distribution.

The fourth section measured the fatigue levels, specifically by investigating the perception of breathlessness, i.e., air hunger, tachycardia, perceived during the pandemic. The mean average score was 6.37 ± 2.89, attesting to an overall high score for this section. The density plot, Figure 1a, presents a left asymmetrical skewness of the data (skewness = −0.48). Additionally, the boxplot, Figure 1b, shows the left whisker longer than the right one, meaning that the distribution tail is longer at left, namely, the students’ fatigue levels distribution is clustered to a high score; therefore, the students did not experience high levels of fatigue due to anti-infection mask usage during COVID-19. 

Secondly, students have been divided into two groups: PA < 150 min/week and PA ≥ 150 min/week, and then sub-divided by the presence of neck pain, low back pain, or both pains. As reported in Table 3, 50.5% of them stated that they experienced pain, and those with neck and low back pain belongs predominantly to the group of individuals practicing PA < 150 min/week. Data suggest that students are more prone to suffer neck pain than low back pain, 43.5%, and 33.5%, respectively. Neck pain is the most frequently reported site of pain for both PA < 150 min/week and PA ≥ 150 min/week groups.

### 3.3. Bodyweight and Seated Time Due to Activities Restriction during One Year of the COVID-19 Pandemic in Italy

A total of 861 students (52.1%) reported an increase in body weight after one year. Specifically, 61.9% reported a bodyweight increase of less than 5 kg, 35.1% a bodyweight increases between 5 kg and 10 kg, and only 3% stated a bodyweight increase over 10 kg. 

Almost all students stated a seated time higher than 4 h/day. The 46.9% reported a seated time between 4 and 8 h, 37.1% between 8 and 12 h, and a small percentage, 8.2%, higher than 12 h.

**Figure 2:**
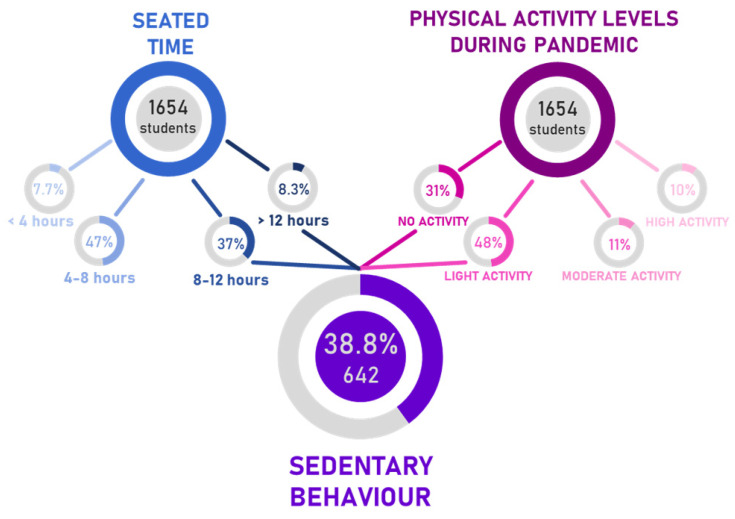
Estimation of sedentary participants depending on seated time and physical activity levels during the pandemic.

Among those participants who reported PA levels lower than 150 min/week together with a seated time >8 h/day (38.8% of total) (Figure 2), 40.8% did not report a bodyweight increase, 34.6% stated a bodyweight increase lesser than 5 kg, 21.5% a bodyweight increase ranged between 5 kg and 10 kg, and 3.1% stated a bodyweight increase over 10 kg. These data suggest a strong correlation between a moderate-to-high body weight increase and PA levels lower than 150 min/week. The odds of weight increase for those showing a sedentary behavior are 5.36 (OR with 95% CI ranging from 3.04 to 9.47) times of those practicing PA, Table A1.

### 3.4. Neck Pain during One Year of COVID-19 Pandemic in Italy

Participants were asked to give information about neck pain onset during the pandemic or if they were used to experience neck pain even before the pandemic; comprehensive data are shown in Table 4. Summarily, 718/1654 students (43.5%) reported the presence of neck pain. Concerning the subjects with pain, 72.1% declared having experienced neck pain during the pandemic period. The pain frequency data are almost equally distributed among all the respective answers. The highest concentration of VDS score is 45.7% moderate and 32.3% mild. Concerning the specific time window of the day, 73.3% experience neck pain after several hours of study, and 60.7% of the pain group state the seated posture as the most pain-arise related. About the relief strategies, 35.4% keep the pain during daily activities until it resolves independently, while 32.9% prefer to perform a specific exercise to reduce it. 77.1% of the latter perform neck muscle stretching and joint mobilization exercises, Table 5. Lastly, all participants were asked to recognize whether the pandemic restrictions had affected the pain onset or not. The 47.6% stated a slight pain increase, whereas 13.4% stated a severe pain increase. Furthermore, 17% of them experienced pain for the first time during the pandemic, 21.2% do not impute the pain onset to the pandemic restriction.

### 3.5. Low Back Pain during One Year of COVID-19 Pandemic in Italy

Participants were asked to give information about low back pain onset during the pandemic or if they were used to experience low back pain even before the pandemic; comprehensive data are shown in Table 4. Summarily, 554/1654 students (33.5%) reported the presence of low back pain. Concerning the subjects with pain, 72.9% declared having experienced low back pain during the pandemic period. The pain frequency data are almost equally distributed among all the respective answers. The highest concentration of VDS score is 39.4% moderate and 35.4% mild. Concerning the specific time window of the day, 69.7% experience low back pain after several hours of study. Furthermore, 50.5% of the pain group states that seated posture is the most pain-arise related, while 30% experience pain when walking, doing PA, or doing housework. About the relief strategies, 37.9% keep the pain during daily activities until it resolves independently, whereas another 37.9% prefer to perform a specific exercise to reduce it. 88.6% of the latter perform muscle stretching and joint mobilization exercises for the back, Table 5. Lastly, all participants were asked to recognize whether the pandemic restrictions had affected the pain onset or not. The 47.3% stated a slight pain increase, whereas 22.4% stated a severe pain increase. Furthermore, 13% of them experienced pain for the first time during the pandemic, 15.5% do not impute the pain onset to the pandemic restriction. 

### 3.6. Neck and Low Back Pain and Sedentary Behavior during One Year of COVID-19 Pandemic in Italy

Neck and low back pain data were then crossed with sedentary conditions. Sedentary behavior (SB) was referred to individuals with PA levels <150 min/week (78.6% of the total of the students) and seated time greater than 8 h/day (45.3% of total the students). Sedentary students result as 38.8% of the total (642/1654). A percentage of 52.3% and 38.9% of them stated to experience neck and low back pain, respectively. These results might suggest that 1 out of 2 students and 1 out of 3 students having SB can be prone to suffer from neck and low back pain, respectively. The OR for the neck pain sample is 1.95 with a 95% CI ranging from 1.44 to 2.64, Table A2. The OR for the low back pain sample is 1.79 with a 95% CI ranging from 1.29 to 2.49, Table A3.

### 3.7. Neck VDS Scores and Pain Frequency Compared to Physical Activity Levels

Data of neck VDS score and pain frequency have been crossed with PA levels to understand if PA can modulate pain perception and occurrence. VDS contingency table, Table 6, shows the highest number of students experiencing pain is concentrated between No PA and Light PA with Mild Pain and Moderate Pain. The highest concentration is in those practicing Light PA and experiencing Moderate Pain, 24.2% of the total. 

Pain frequency contingency table, Table 7, shows the frequency of neck pain during the month is almost equally shared among all the groups, except for the 16 times/month group, which comprises the lowest number of individuals. The reported percentages show that the highest concentration is in those practicing Light PA and experiencing the pain two times/month, 16.7% of the total.

**Figure 3:**
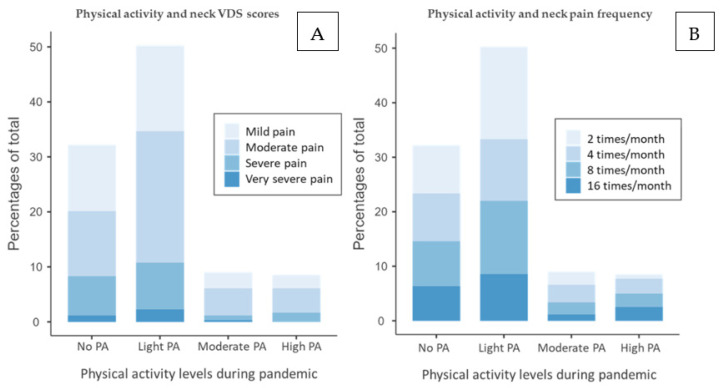
(**A**) Stacked bar of VDS neck score and physical activity levels and (**B**) stacked bar of pain frequency and physical activity levels. No PA: no physical activity, Light PA: physical activity <140 min/week, Moderate PA: ≃150 min/week, High PA: >200 min/week.

The stacked bar of neck pain shows the correlation between PA levels, VDS score (Figure 3A), and pain frequency (Figure 3B). Students belonging to the No PA and Light PA categories represent most subjects with pain, suggesting that those practicing PA < 150 min/week (No PA and Light PA subjects) are more prone to experience pain. Secondly, those with a VDS score = Severe pain or very severe pain are expressed as a higher percentage in groups No PA and Light PA. Among subjects with PA ≥ 150 min/week (Moderate and High PA), the higher percentages of VDS score are represented by mild and moderate VDS levels of pain. These results suggest a lower existence of students with pain and, in addition, lower pain perception among those who comply with WHO guidelines. Similarly, the pain frequency stacked bar, Figure 3B, shows a similar trend. Nevertheless, a notable percentage of those experiencing pain 16 times/month in the High PA group suggest a possible relationship between pain frequency and high activity levels. Notably, the population suffering from neck pain is more concentrated in the Light PA group for both plots. 

### 3.8. Low back VDS Scores and Pain Frequency Compared to Physical Activity Levels

Data of low back VDS score and pain frequency have been crossed with PA levels to understand if PA can modulate pain perception and occurrence. VDS contingency table, Table 8, shows the highest number of students experiencing pain is concentrated between No PA and Light PA with Mild Pain and Moderate Pain. The highest concentration is in those practicing Light PA and experiencing Mild Pain, 21.3% of the total. 

Pain frequency contingency table, Table 9, shows the frequency of low back pain during the month is almost equally shared among all the groups, slightly higher for the 8 times/month group. The reported percentages show that the highest concentration is in those practicing Light PA and experiencing pain 8 times/month, 16.6% of the total. 

**Figure 4:**
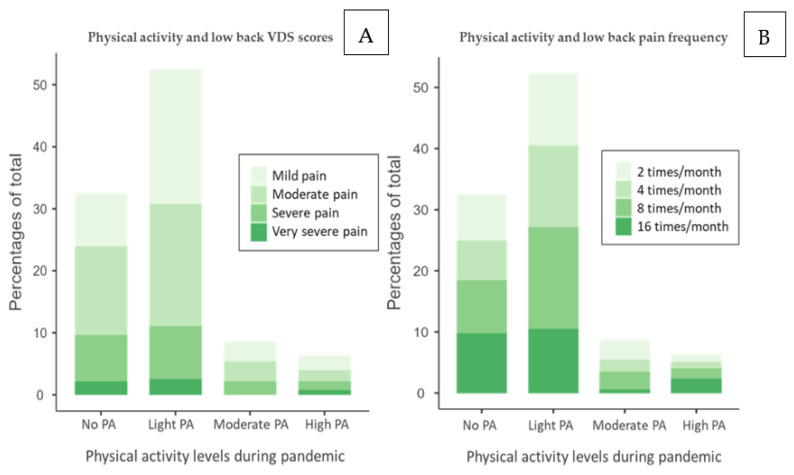
(**A**) Stacked bar of VDS low back score and physical activity levels and (**B**) stacked bar of pain frequency and physical activity levels. No PA: no physical activity, Light PA: physical activity <140 min/week, Moderate PA: ≃150 min/week, High PA: >200 min/week.

The stacked bar of low back pain shows the correlation between PA levels, VDS score, Figure 4A, and pain frequency, Figure 4B. Its trend is in accordance with the neck pain stacked bar. Students belonging to No PA and Light PA categories represent most subjects with pain, suggesting that those with PA < 150 min/week are more prone to experience pain. Secondly, those with a VDS score resulting from severe pain or very severe pain are mainly present in the No PA and Light PA groups. The group Moderate PA shows a similar trend for all pain levels, although very severe pain is not present. This data might suggest that PA level close to 150 min/week, i.e., Moderate PA, is associated with lower pain perception, while PA levels lower or higher than WHO guidelines might determine an increase in pain perception. Similarly, the pain frequency stacked bar, Figure 4b, shows a similar trend. Nevertheless, there is a notable percentage of those experiencing pain 16 times/month and 8 times/month in the No PA and Light PA groups, suggesting a possible relationship between pain frequency and low activity levels. A considerable percentage of those with 16 times/month pain presence is also represented in the High PA group. This trend is similar to the VDS stacked bar and might suggest that PA levels close to 150 min/week, Moderate PA, are associated with a lower pain frequency, while PA levels lower or higher than WHO guidelines might determine an increase in pain frequency. Notably, the population suffering from low back pain is more concentrated in the Light PA group for both plots.

## 4. Discussion

Imposed quarantine due to the COVID-19 pandemic imposed a severe reduction in daily activities and inevitably increased the onset of MP. In the available literature concerning the COVID-19 aspects, the relationship between PA reduction and MP has not yet been investigated. To the best of our knowledge, this is the first study discussing the relationship between the reduction of PA levels, SB increase, and MP onset in university students after one year of COVID-19 restrictions in Italy. 

**Figure 5:**
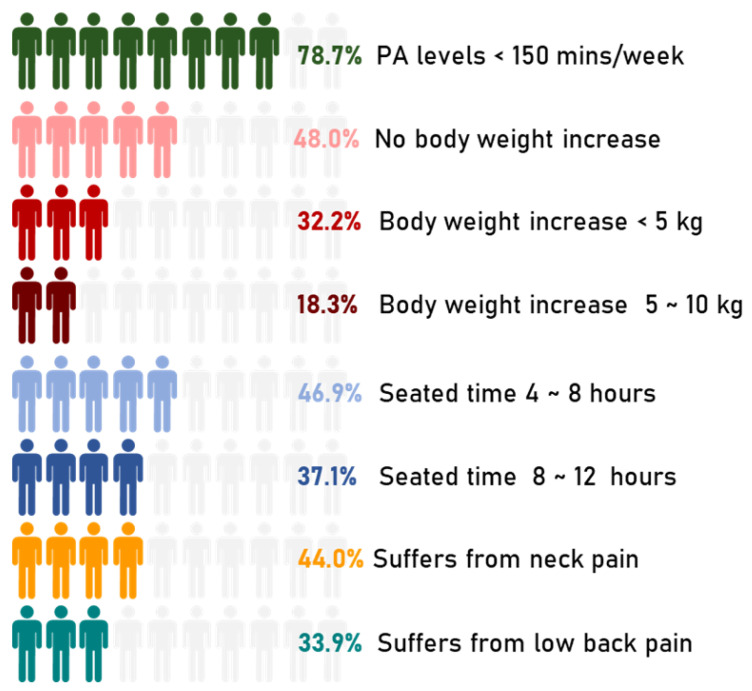
Primary outcomes of the present study. The representation is counted as *n*/10 based on the respective data.

The disease outbreak has changed young people’s lives who were used to spending most of the day away from home between study, work, commitments, friends, sports, and entertainment. Figure 5 shows the primary outcomes of this study. 

**Figure 6:**
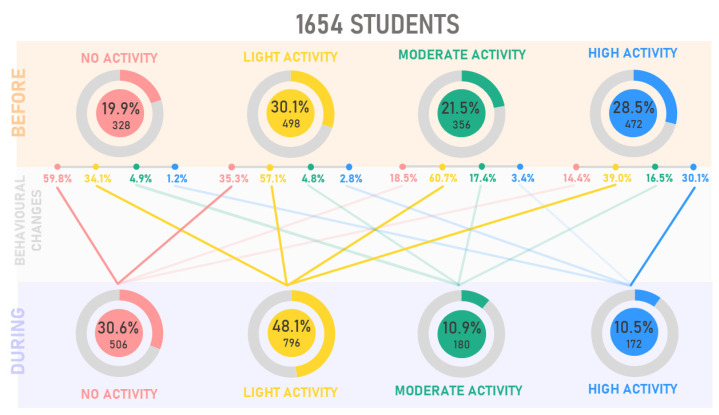
Physical activity changes before (March 2020) and during (March 2020/2021) one year of pandemic restrictions.

During the first pandemic period, March-May 2020, sport-related public facilities were closed, making it challenging to practice jogging, running, or walking long distances. Maugeri et al. [17] conducted an epidemiological analysis during the first quarantine period on 2524 Italian subjects aged 18 and 70. PA levels suffered a moderate decrease, those who practiced moderate activity decreased by about 6%, while those who practiced intense activity by 11%. Some sports activities resumed from June but starting from October, with the increase in the number of infections, sports centers closed once more, and the red zones blocked the students at home again. This event did not permit the expected recovery of sports activities, so according to our data, about 30% of the participants did not return to the PA levels they had before the pandemic after one year of experiencing a pandemic. Figure 6 shows how PA levels have changed between before and during the pandemic in one year. About 60% of those inactive before the pandemic did not change this behavior during the pandemic, while 35% practicing light activity before the pandemic became inactive during the pandemic. Interestingly, the highest percentage of people for each group, except for the inactive ones, is channeled into the light activity group. The increase of subjects performing light levels PA is due to the presence of those who started practicing PA (14.1%), probably to overcome the severe limitations of daily activities, and those who reduced their PA levels from moderate and high to light (27.1% and 23.1%, respectively). Among those who did not practice PA before the pandemic (19.9%), 40.2% started practicing PA during the pandemic. These findings contrast with Hall et al. [18], are speculating that those already sedentary before the pandemic would hardly increase their PA levels during the pandemic. Several studies analyzed reduced PA and SB worldwide [19,20,21,22]. Many authors decreed a possible end of these conditions in the summer of 2020, although unfortunately, starting from October 2020, the severe limitations were back in effect in Italy. WHO guidelines of PA and SB [23] recommend doing at least 150 min of moderate-intensity PA throughout the week and limiting sedentary time. In line with these guidelines, the students who reported MP were divided into two groups based on PA adherence. The data show a high prevalence of students reporting pain in the PA < 150 min/week group, representing 41.7% versus 8.8% of those performing PA ≥ 150 min/week.

The 72.2% of those with neck pain experienced it during the pandemic and mainly due to several hours of study, as expected since they had to attend courses through electronic devices due to the restriction measures. As reported by Mowatt et al. [24], the most frequent health problems among those using electronic devices for several hours are computer vision syndrome, neck, shoulder, and back pain, and specifically, 89.9% of undergraduate university students have a prevalence of these health problems [25]. Prolonged use of mobile phones, tablets, or laptops to attend online lessons or to spend time on social media may negatively affect neck and shoulders pain [26]. This relationship, anyhow, has been validated by several epidemiological studies, which confirmed that assuming a wrong posture for many hours and having SB is strongly related to the severity of neck pain [27,28,29,30,31]. Our data show how the levels of VDS score, and frequency of neck pain onset are considerably lower for students performing moderate to high PA levels compared to those with light or no PA levels. These findings confirm the hypothesis of a greater likelihood of having neck pain for those with low levels of PA as highlighted by Scarabottolo et al. [32] and Guddal et al. [33]. Conversely, we strongly disagree with Sitthipornvorakul et al. [34], who assert strong evidence of no association between PA and neck pain.

The 72.9% of the students with low back pain experienced it during the pandemic, mainly due to several hours of study, probably related to a wrong posture assumed during it, as for the neck pain. Stressors, fear of pain, and lack of PA, according to Amelot et al. [35], are the most critical factors affecting LBP occurrence. Only PA levels have been evaluated in the present study, but psychosocial repercussions over mental health were present in line with another analysis conducted among the Italian population [36]. As for neck pain, VDS score and frequency for low back pain was considerably lower for students with moderate to high PA levels, compared to those with light or no PA. These results are in line with Wedderkopp et al. [36], where physically active students had a low predisposition to experiencing back pain. Likewise, Guddal et al. [33] observed that moderate levels of PA were correlated to reduced LBP onset. However, as we highlighted in the results section, excessively high PA levels might increase the risk of spinal pain because intense activities might contribute to a wrong posture and lead to pain onset [37]. 

Two studies [38,39] pointed the absence of correlation between a sedentary lifestyle and the occurrence of MP in medical students with LBP. Our data contrast these authors’ points of view because the OR between SB and low back pain was 1.79 (95% CI, 1.29–2.49), Table A2. However, these authors did not clearly state what describes SB. While Moroder [38] did not classify sitting time and PA levels threshold as sedentary, Chen S.M. [39] considered only time spent sitting, omitting the PA levels. Even if this topic is still debated among the scientific community, our findings strongly agree with a recent meta-analysis published in Nature journal by Alzahrani et al. [40], who highlighted the importance of medium to moderate PA levels to decrease the risk of LBP. Furthermore, epidemiological research has shown that assuming a wrong posture for a prolonged time, sitting for many hours, or simply taking part in SB are strong predictors of adverse health outcomes such as cardiovascular diseases [41], diabetes [42], cancer [43], musculoskeletal pain [44], or depression [45]. In line with our investigations about SB and MP presence, Shrier and Feldman [46,47] identified the prolonged sitting position as a prevalent risk factor for MP onset. In general, it can be assumed that the more frequent students are physically inactive during the week, the more frequent is the risk of suffering from chronic pain [48].

Concerning the pain relief strategies, Mimi Mun Yee Tse et al. [49] conducted a similar study of university students in Hong Kong and stated a high percentage of them adopting pharmacological methods to contrast MP. This condition differs from our data, as our sample’s highest pain relief strategy was performing physical exercises or waiting until the pain resolves. Those experiencing pain should prefer non-pharmacological treatment initially, favoring exercises or rehabilitation protocols [50] instead. Therefore, education for the young is needed to give them resources to manage their condition, such as exercises sheets, pain management guidelines, or prevention methods [50,51].

Further studies are needed to understand the aspects related to a sedentary lifestyle and pain. What is clear is that we must work on two fronts since, following this trend, the 2025 global PA target (10% reduction of physical inactivity) will not be met [52]. Firstly, National governments should develop new approaches to engage the unwilling population to increase or start PA programs, especially after one year of restrictions. Secondly, there is a need to understand the reasons behind some students’ indifference towards PA, since, during the pandemic, they could have trained at home to counterbalance psychological and physiological distress [53,54]. The last decade’s general increase of physical inactivity prompted the WHO in 2018 to provide a plan until 2030 to encourage the world population to be more active [55]. The aim is to invest in policies to promote sports activities, jogging, or just recreational activities to achieve different sustainable development goals by 2030. Trivial as it may seem, yet simply carrying out a student’s daily activities such as leaving home in the morning, going to university, walking with friends, or visiting a shop can be worth it to maintain an active body, and thus avoid the onset of pain [56]. In line with WHO guidelines, we suggest the need to plan educational programs that encourage students to practice exercise. For instance, with the help of professionals, e.g., kinesiologists, universities could plan a 10-min break within lessons to perform simple exercises to keep the body active and avoid the classic pains from incorrect posture. 

This study has some limitations that need to be considered in the results’ interpretation. First, the questionnaire was administered through online channels, which may determine a disinterest in answering all questions carefully. Second, it was a self-reported questionnaire, indicating an underestimation or overestimation of the self-conditions based on the questions. Third, a bias regarding PA levels before the pandemic may be present due to the time elapsed. Fourth, this study has a cross-sectional design, so inference must be evaluated carefully. Conversely, many responses, the presence of different check-questions useful to reduce the bias, the strong consistency due to a close age range, and the reduced likelihood of having other conditions that could lead to MP enhance the study’s strength.

In terms of future research, we expect to conduct further research like this after recovering entirely from the COVID-19 pandemic to determine if PA and SB levels retrieved after this social catastrophe and investigate how MPs can be modulated through daily exercises.

## 5. Conclusions

One year of COVID-19 restrictions forced the students to reduce their daily activities and triggered, in some cases, adverse health outcomes. An overall reduction of physical activity and musculoskeletal pain onset was observed, especially for those who did not respect the WHO physical activity guidelines. These findings highlight the alarming condition of the presence of musculoskeletal pain in a young population. Universities are called upon to handle this situation in the best possible way; a preventive approach is required as a young person experiencing pain today could be an adult with chronic pathologies tomorrow, leading to earlier habitual use of drugs, namely, which is a burden to public health.

## Figures and Tables

**Table 1:** Characteristics of participants.

	(n)	(%)
Participants	165	
Female	1026	62
Male	628	38
Underweight (BMI)	130	7.9
Normal weight (BMI)	1176	71.1
Overweight (BMI)	348	21

n: number, %: percentage, BMI: Body mass index.

**Table 2:** Physical activity levels before and during the pandemic.

	Before the Pandemic		During the Pandemic		
	(n)	(%)	(n)	(%)	*p*-value *
Absent	328	19.9	506	30.6	<0.001
Light	498	30.1	796	48.1	
Moderate	356	21.5	180	10.9	
High	472	28.5	172	10.5	

n: number; %: percentage, * according to chi-square test.

**Table 3:** Students with musculoskeletal pai divided by physical activity levels.

	Total		Neck Pain		Only Neck Pain		Both Pains		Low Back Pain		Only Low Back Pain		
	(n)	(%)	(n)	(%)	(n)	(%)	(n)	(%)	(n)	(%)	(n)	(%)	*p*-value *
PA < 150	680	41.7	590	35.7	216	13.1	292	17.6	470	28.4	172	10.4	0.0184
PA ≥ 150	146	8.8	128	7.8	66	4	50	3	84	5	30	1.8	

PA < 150: physical activity lesser than 150 min/week; PA ≥ 150: physical activity at least 150 min/week; n: number; %: percentage based on the whole population, * according to chi-square test.

**Table 4:** Overall characteristics of musculoskeletal pains.

	Neck Pain Students (718)		Low Back Pain Students (554)	
	(n)	(%)	(n)	(%)
**Temporal window**				
Pain within the last 4 months	400	55.7	260	46.9
Pain within the last 9 months	118	16.4	144	26.0
Pain within the last 12 months	200	27.9	150	27.1
**Frequency**				
16 times/month	132	18.4	130	23.5
8 times/month	192	26.7	166	30.0
4 times/month	188	26.2	124	22.3
2 times/month	206	28.7	134	24.2
**Pain intensity**				
0—no pain	0	0	0	0
1—mild pain	232	32.3	196	35.4
2—moderate pain	328	45.7	218	39.1
3—severe pain	132	18.4	110	19.9
4—very severe pain	26	3.6	30	5.4
5—worst pain ever	0	0	0	0
**Daytime window**				
After several hours of study	526	73.3	386	69.7
After waking up in the morning	84	11.7	54	9.7
In the late evening	66	9.2	62	11.2
No specific moment	42	5.8	52	9.4
**Pain posture onset**				
Sitting	436	60.7	280	50.5
Walking/housework	64	8.9	166	30.0
No specific circumstance	218	30.4	108	19.5
**Pain relief strategy**				
Performance exercises	236	32.9	210	37.9
Medicines	130	18.1	46	8.3
Sleep	98	13.6	88	15.9
Wait until it resolves	254	35.4	210	37.9

**Table 5:** Exercise classification of those choosing exercises as pain relief strategy.

	Neck Pain Students (236)		Low Back Pain Students (210)	
	(n)	(%)	(n)	(%)
Stretching and joint mobilization	182	77.1	186	88.6
Performing sport activities	16	6.8	8	3.8
Counter-resistance/isometric	38	16.1	16	7.6

**Table 6:** Contingency table of physical activity levels and VDS neck scores.

	VDS Levels											
	Mild Pain		Moderate Pain		Severe Pain		Very Severe Pain		Total		χ^2^ *	τb **
	(n)	(%)	(n)	(%)	(n)	(%)	(n)	(%)	(n)	(%)	0.020	−0.002
No PA	82	11.4	86	12	52	7.2	8	1.1	228	31.8		
Light PA	110	15.3	174	24.2	62	8.6	16	2.2	362	50.4		
Moderate PA	22	3.1	33	4.6	5	0.7	6	0.8	66	9.2		
High PA	18	2.5	32	4.5	12	1.7	0	0	62	8.6		
Total	232	32.3	325	45.3	131	18.2	30	4.2	718	100		

n: number; %: percentage, No PA: no physical activity; Light PA: <150 min/week of MVPA, Moderate PA: ≃150 min/week of MVPA; High PA: >200 min/week of MVPA; VDS: verbal descriptive scale; * Chi-square *p*-value; ** Kendall-tau value.

**Table 7:** Contingency table of physical activity levels and neck pain frequency.

	Monthly Frequencies											
	2 Times/Month		4 Times/Month		8 Times/Month		16 Times/Month		Total		χ^2^ *	τb **
	(n)	(%)	(n)	(%)	(n)	(%)	(n)	(%)	(n)	(%)	0.010	0.02
No PA	62	8.6	62	8.6	60	8.4	44	6.1	228	31.8		
Light PA	120	16.7	82	11.4	98	13.6	62	8.6	362	50.4		
Moderate PA	18	2.5	24	3.3	16	2.2	8	1.1	66	9.2		
High PA	6	0.8	20	2.8	18	2.5	18	2.5	62	8.6		
Total	206	28.7	188	26.2	192	26.7	132	18.4	718	100		

n: number; %: percentage, No PA: no physical activity; Light PA: <150 min/week of MVPA, Moderate PA: ≃150 min/week of MVPA; High PA: >200 min/week of MVPA; * Chi-square *p*-value; ** Kendall-tau value.

**Table 8:** Contingency table of physical activity levels and VDS low back scores.

	VDS Levels											
	Mild Pain		Moderate Pain		Severe Pain		Very Severe Pain		Total		χ^2^ *	τb **
	(n)	(%)	(n)	(%)	(n)	(%)	(n)	(%)	(n)	(%)	0.026	−0.09
No PA	46	8.3	80	14.4	42	7.6	12	2.2	180	32.5		
Light PA	118	21.3	110	19.9	48	8.7	14	2.5	290	52.3		
Moderate PA	18	3.2	18	3.2	12	2.2	0	0	48	8.7		
High PA	14	2.5	10	1.8	8	1.4	4	0.7	36	6.5		
Total	196	35.4	218	39.4	110	19.9	30	5.4	554			

n: number; %: percentage, No PA: no physical activity; Light PA: <150 min/week of MVPA, Moderate PA: ≃150 min/week of MVPA; High PA: >200 min/week of MVPA; VDS: verbal descriptive scale; * Chi-square *p*-value; ** Kendall-tau value.

**Table 9:** Contingency table of physical activity levels and low back pain frequency.

	Monthly Frequencies											
	2 Times/Month		4 Times/Month		8 Times/Month		16 Times/Month		Total		χ^2^ *	τb **
	(n)	(%)	(n)	(%)	(n)	(%)	(n)	(%)	(n)	(%)	0.037	−0.05
No PA	42	7.6	36	6.5	48	8.7	54	9.7	180	32.5		
Light PA	66	11.9	74	13.4	92	16.6	58	10.5	290	52.3		
Moderate PA	18	3.2	8	1.4	16	2.9	6	1.1	48	8.7		
High PA	8	1.4	6	1.1	9	1.6	13	2.3	36	6.5		
Total	134	24.2	124	22.4	165	29.8	131	23.6	554			

n: number; %: percentage, No PA: no physical activity; Light PA: <150 min/week of MVPA, Moderate PA: ≃150 min/week of MVPA; High PA: >200 min/week of MVPA; * Chi-square *p*-value; ** Kendall-tau value.

## Data Availability

The data presented in this study are available on request from the corresponding author.

## Appendix A

**Table A1:** Odds ratio between sedentary behavior and body weight increase.

	Weight Increase	No Weight Increase	Total	Odds Ratio	τb *
Sedentary	158	484	642	5.36 (3.04–9.47)	−0.213
No sedentary	14	230	244		
Total	172	714	886		

Sedentary: physical activity levels < 150 min/week and seated time > 8 h/day; * Kendall-tau value.

**Table A2:** Odds ratio between sedentary behavior and low back pain.

	Low Back Pain	No Low Back Pain	Total	Odds Ratio	τb *
Sedentary	250	392	642	1.79 (1.29–2.49)	0.118
No sedentary	64	180	244		
Total	314	572	886		

Sedentary: physical activity levels < 150 min/week and seated time > 8 h/day; * Kendall-tau value.

**Table A3:** Odds ratio between sedentary behavior and neck pain.

	Neck Pain	No Neck Pain	Total	Odds Ratio	τb *
Sedentary	336	306	642	1.95 (1.44–2.64)	−0.145
No sedentary	88	156	244		
Total	424	462	886		

Sedentary: physical activity levels < 150 min/week and seated time > 8 h/day; * Kendall-tau value.
